# Complex Pathological Femoral Fracture in a Multiple Myeloma Patient Undergoing Intertrochanteric Fixation: A Case Report

**DOI:** 10.7759/cureus.58224

**Published:** 2024-04-14

**Authors:** Ameer Khan, Daniel K Kyeremateng, Zeeshan A Khan, Muhammad S Tariq, Munir Khan

**Affiliations:** 1 Cardiology, Tameside General Hospital, Ashton-under-Lyne, GBR; 2 Medicine, University of Leeds, Leeds, GBR; 3 Orthopaedics, Tameside General Hospital, Ashton-under-Lyne, GBR; 4 Orthopaedics and Trauma, North Manchester General Hospital, Manchester, GBR; 5 Orthopaedics and Trauma, Tameside General Hospital, Ashton-under-Lyne, GBR; 6 Internal Medicine, Tameside General Hospital, Ashton-under-Lyne, GBR; 7 Orthopedics and Traumatology, Tameside General Hospital, Ashton-under-Lyne, GBR

**Keywords:** metastatic bone lesions, holistic care for patients, gamma nail, pathological femur fracture, myeloma

## Abstract

Pathological fractures commonly occur in patients with metastatic bone diseases, particularly multiple myeloma. The current optimal management for metastatic pathological lesions affecting the proximal femur is surgical intervention. Surgical planning and appropriate use of imaging modalities are pivotal in the appropriate treatment of pathological fractures. Impending fractures create added layers of complexity in the decision-making process. The appropriateness of different surgical interventions involves a multi-disciplinary approach and the importance of holistic healthcare is paramount in these circumstances.

## Introduction

The appendicular skeleton's most common site for metastatic bone disease is the proximal femur, with associated pathological fractures in the peri trochanteric region [1]. This targeting of the appendicular skeleton by metastatic bone disease has a significant impact on cancer-related morbidity and mortality. The proximal femur is subject to the highest compressive and tensile loads in the body, reaching a maximum of up to 1200 pounds per square inch [2]. Both cortical and trabecular bones are affected, disrupting their structure and strength due to metastasis [3]. Subsequently, the risk of pathological fractures significantly increases with increased bone loading during activities of daily living, leading to increased morbidity and mortality rates [4].

The identification and treatment of potential pathological fractures in femurs affected with metastatic lesions represents an area of research interest. Metastases commonly manifest in the long bones, often presenting with symptoms of pain and pathological fractures, thereby necessitating intervention such as radiotherapy or surgery [1]. Surgical treatment options include plates or intramedullary nails depending upon the location of the lesion, the extent of bone involvement, and expected survival rates [1]. The use of surgical modalities is dictated by the site of the lesion, the extent of bone involvement, and the patient's prognosis.

In this report, we discuss the case of a multiple myeloma patient presenting with a pathological fracture in the intertrochanteric region of the femur. This fracture is concomitant with a metastatic lesion in the midshaft of the same femur, highlighting the need for a comprehensive surgical approach. The significance of this case lies not only in addressing the immediate fracture but also in managing the metastatic lesion, posing multifaceted challenges when developing a treatment plan. This case underscores the significance of employing a holistic diagnostic approach, particularly in the context of metastatic bone lesions, to ensure comprehensive assessment and optimal treatment planning.

## Case presentation

A male in his 80s, living independently with his wife, presented with acute onset left hip pain accompanied by an audible crack while moving from his bed to the commode. This was associated with an inability to bear weight on the left leg. On physical examination, there was tenderness around the greater trochanter, left leg shortening, and external rotation, alongside an inability to straight leg raise. The distal neurovascular status in the left leg remained intact. The patient was wheelchair-bound and lived with his wife at home. He reported no history of smoking or alcohol use but was allergic to isosorbide mononitrate and amlodipine.

The patient's medical history consists of a range of conditions, notably multiple myeloma diagnosed in 2015, which required chemotherapy. Subsequent relapses in 2017 and 2018 required different treatments due to the progression of his disease. Additionally, the patient had various comorbidities, including atrial fibrillation managed with a permanent pacemaker, stage IV chronic kidney disease, diverticular disease, congestive cardiac failure, ischemic heart disease, cataracts, oesophageal dysmotility and sarcoidosis. The patient has a previous surgical history of a left knee replacement and fracture management with intermedullary nails for his right femur.

Given the patient's medical history included a diagnosis of multiple myeloma, it was essential to complete a thorough diagnostic workup. Preoperative blood tests revealed a low haemoglobin level indicative of anaemia, while kidney function tests showed elevated urea, creatinine, and reduced estimated glomerular filtration rate (eGFR) due to chronic kidney disease stage IV. Electrolyte levels were within normal limits, except for the aforementioned renal function abnormalities. Haemostasis evaluation via bleeding and clotting profile yielded normal results. The bone profile exhibited hypercalcemia and elevated alkaline phosphatase levels, suggestive of increased bone resorption. Despite the patient's American Society of Anesthesiologists (ASA) 4 status, ECG and chest X-rays revealed no remarkable findings. Additionally, a full blood count revealed a normal blood profile except for the low haemoglobin level indicative of anaemia.

**Figure 1:**
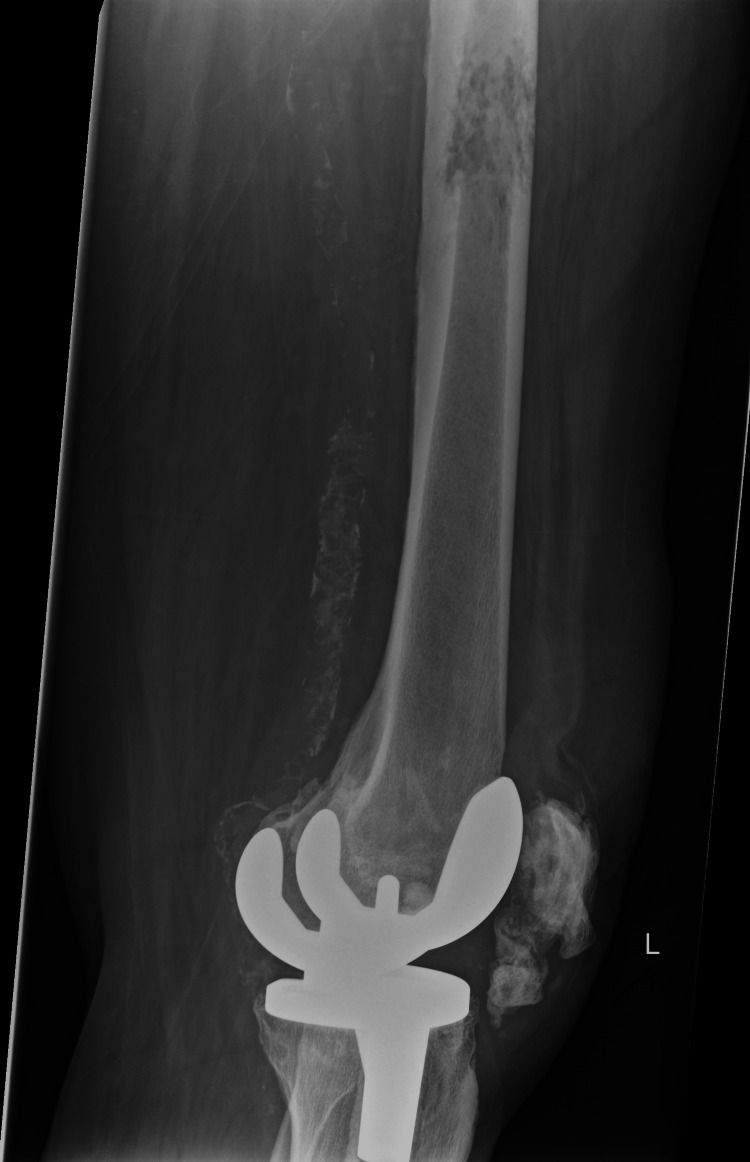
Region of patchy lucent bone is demonstrated to the proximal third of the left femoral shaft, indicating an impending fracture

**Figure 2:**
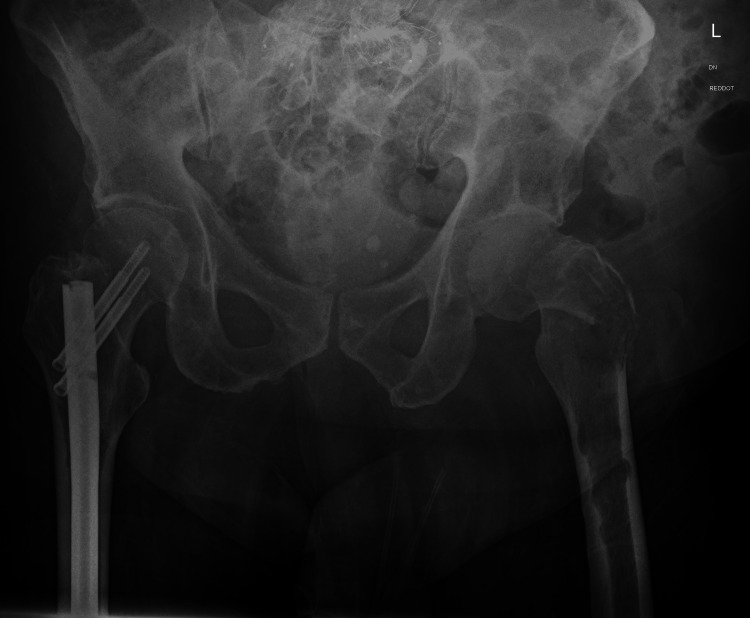
A pathological intertrochanteric fracture through the left proximal femur and previous intermedullary nailing to the right femur

X-ray imaging of the left hip and whole femur depicted an intertrochanteric fracture with an osteolytic lesion and an additional lesion involving the midshaft of the femur, while no clear lesion was evident in the distal femur (Figures 1, 2)

Due to an incompatible pacemaker in situ, MRI scanning was contraindicated. When balancing the findings from the whole case, the primary differentials included an intertrochanteric fracture hip, osteoarthritis hip, and septic arthritis of the hip.

In this case, the diagnosis was confirmed at a very early stage due to the presence of clinical symptoms and plain film findings align with the features of an intertrochanteric hip fracture with the addition of a lesion within the midshaft of the femur (Figures 1, 2).

Initial fracture management, as per the Advanced Trauma Life Support (ATLS) guidelines, involved stabilizing the fracture, providing adequate analgesia, and maintaining close monitoring of the patient's clinical condition [5]. Pain control involved the use of fascia iliaca compartment blocks. Venous thromboembolic prophylaxis was administered as per hospital trust guidance, advocating the use of low molecular weight heparin as the standard prophylactic treatment in fractures of the pelvis, hip, and femur. Notably, despite being a Jehovah's Witness, the patient provided special consent to accept blood transfusions if deemed necessary for survival.

During the preoperative planning multi-disciplinary meeting, it was decided to use a long gamma nail instead of a short gamma nail, primarily due to the identification of an impending fracture, with a Mirel's score >8 located at the midshaft of the femur. The patient was prepared for spinal anaesthesia, and a prophylactic dose of intravenous antibiotics was administered accordingly.

**Figure 3:**
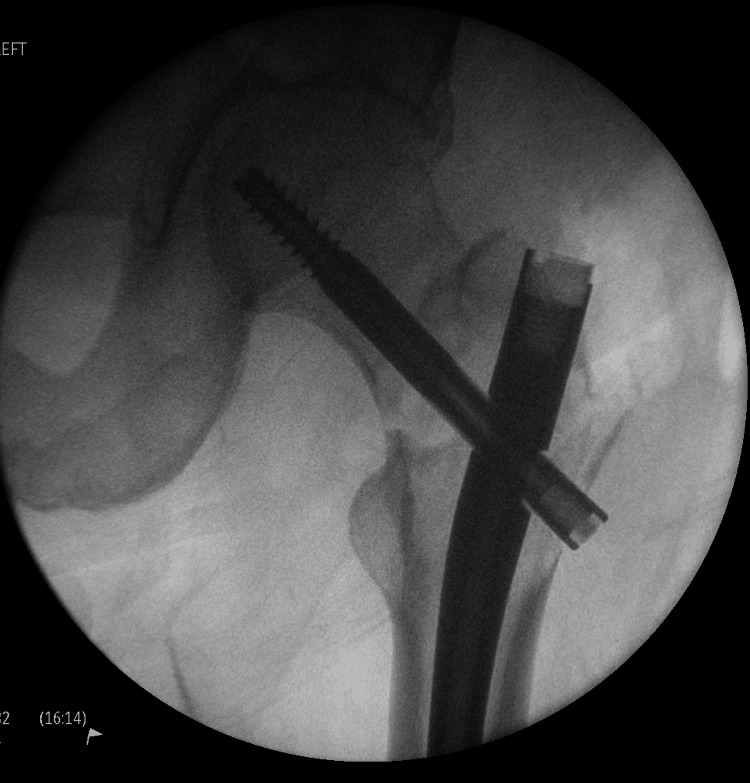
Fluoroscopic image of the left proximal femur with long gamma nail insertion

**Figure 4:**
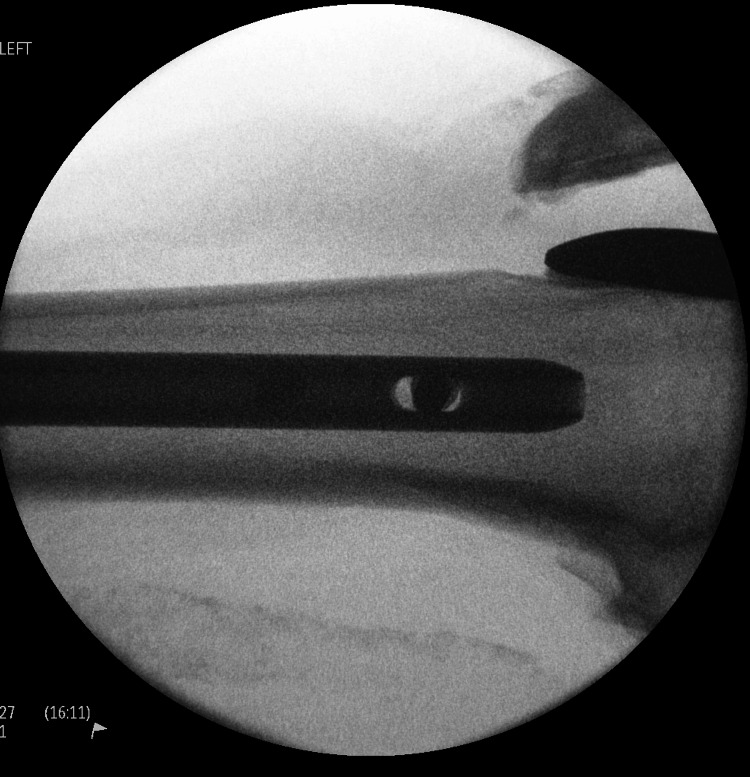
Fluoroscopic imaging of left distal femur with long gamma nail insertion

In the operating theatre, the procedure involved a closed reduction and long intermedullary gamma nailing of the left femur fracture (Figures 3, 4).

During the operation, a biopsy of the bone ream was taken for subsequent histological examination. Subsequent to fracture reduction, a longitudinal incision was meticulously made at the greater trochanter. This facilitated the insertion of the intermedullary long gamma nail into the femoral medullary canal over a guidewire and secured with distal and proximal screws. Haemostasis was secured, and the wound was closed with layered sutures, skin clips, and sterile dressings applied. 

Postoperatively, the patient received two doses of intravenous antibiotics and underwent deep vein thrombosis prophylaxis for 35 days as per hospital protocols. Mobilization with full weight-bearing was started under the guidance of the physiotherapists. Removal of skin clips occurred at two weeks postoperatively, with satisfactory wound healing observed. Histological analysis of bone reaming specimens confirmed the presence of multiple large aggregates of mature plasma cells consistent with multiple myeloma, revealing a complication beyond the initial surgical scope.

During the postoperative period, the patient tested positive for COVID-19 but remained asymptomatic without requiring medical intervention or supplemental oxygen. The patient had a course of chemotherapy due, which was administered 25 days postoperatively. The patient remained admitted in hospital throughout the postoperative follow-up period of three months due to a combination of social and medical issues. Postoperative physiotherapy facilitated mobility, with the provision of hoists for transfers between bed, chair, and commode until discharge to an intermediate rehabilitation facility from the medical ward. Follow-up duration adhered to National Hip Fracture Database recommendations, ensuring a minimum of 120 days of monitoring [6].

## Discussion

Pathological fracture management requires the need for complex surgical analysation, involving evaluation of the general health of the patient, the local status of the bone and the extent of metastatic spread [1]. Pathological fractures of the femur are common occurrences, with over 75% of these fractures involving the proximal aspect [1]. A study investigating the post-mortem examination of patients who died from metastatic disease showed that metastases affected the vertebral column 68% of the time, the pelvic bones 48%, and the femur 25% of the time [7].

Three problems were found in our case: a pathological fracture of the intertrochanteric femur, an imminent pathological lesion in the diaphysis of the same femur, and the existence of an implant from a prior knee replacement surgery at the distal femur, which made implant selection extremely important. Consequently, there were two choices available for the surgical planning of fixation: using an intramedullary nail that was long enough to penetrate through the diaphyseal lesion and secure above the knee implant or placing a separate plate for the diaphysis. In order to treat the intertrochanteric fracture, a long gamma nail (LGN) was used. This allowed for both safe locking above the knee implant in the distal femoral condyle and preventive stabilisation of the approaching diaphyseal lesion [8]. Therefore, these factors served as the foundation for the selection of the appropriate implant and surgical technique to successfully address these problems.

For long bone pathological fractures, there are three main therapeutic options: plating, prosthetic reconstruction, or intramedullary (IM) nailing [1]. Benefits of IM nailing include protection of a lengthy bone segment, preservation of the periosteal blood supply due to minimum dissection, and rigid fixation made possible by the interlocking screws on the proximal and distal ends. Distal locking further improves rotational stability and guards against fixation failure, and a big proximal lag screw strengthens the design. IM nails, however, act as load-sharing devices and could pose a problem [1].

The use of either a short gamma nail (SGN) or an LGN is contingent upon various parameters like fracture site, patient characteristics and underlying conditions. LGN provides superior biomechanical support, especially for intertrochanteric fractures in patients with compromised bone integrity, such as in this case. The increased length promotes fracture healing and reduces the risk of implant failure [9]. The surgical conundrum over whether to use LGN versus SGN has long been noted, with historical data suggesting SGN was associated with a higher fracture rate [10]. Studies have shown that the use of LGN increases stability and is favourable in elderly patients [10]. Zhi et al. noted clinically significant hip pain and treatment failure rates when LGNs were used against SGNs in their study [10]. In this case, LGN was used to address the fracture's requirement for strong fixation amidst compromised bone quality and difficulties associated with the treatment of a pathological fracture [9,10].

When faced with co-morbid patients, it is essential that the disease burden of the patient is truly understood before any treatment options are considered. Holistic patient-centred care is vital in determining a treatment strategy for the patient. This highlights the importance of a multi-disciplinary approach to these scenarios, where if it is judged that short-term survival is unlikely, conservative minimally invasive measures should be considered [1].

Appropriate risk stratification is essential in managing complex cases such as this. Mirel's criteria serve as a widely utilised method for estimating fracture risk in impending pathologic lesions in long bones [3]. Impending pathological lesions must be meticulously evaluated to aid in pain relief and functional improvement, and to allow timely surgical intervention [11]. The diagnostic workup includes investigations aimed at characterising the lesion and determining the primary source of metastasis [12]. Factors like pain, location, size, and features of the lesion all increase the likelihood of an imminent fracture [13, 14]. However, because these indicators are retroactive in nature, their predictive power is constrained.

Further research to improve the risk stratification process has revealed that axial cortex involvement greater than 30 mm and circumferential cortical involvement greater than 50% are predictive factors for fracture [11]. While a CT scan is useful for estimating the future risk of a fracture in this population, standard radiographs can also be used to assess axial cortical involvement. This is especially important when advanced imaging modalities, like an MRI or CT scan, are contraindicated because of multiple comorbidities [15].

## Conclusions

Early identification of impending pathological fractures cannot be overstated. A thorough evaluation of the entire bone is necessary to rule out any additional pathology, especially in known cases of metastatic disease. Preoperative surgical planning, in particular the type of implant selected, is crucial, especially in these at-risk patients. Every effort should be made to utilise implants that prioritise stability and facilitate early mobilisation, thereby optimising patient outcomes.

The involvement of a multidisciplinary team is indispensable in the management of patients with multiple comorbidities, especially in cases of metastatic bone disease. Collaboration among these professionals is necessary for addressing the complex needs of patients and optimising their outcomes.
